# Optical Structural Analysis of Individual α‐Synuclein Oligomers

**DOI:** 10.1002/anie.201710779

**Published:** 2018-03-23

**Authors:** Juan A. Varela, Margarida Rodrigues, Suman De, Patrick Flagmeier, Sonia Gandhi, Christopher M. Dobson, David Klenerman, Steven F. Lee

**Affiliations:** ^1^ Department of Chemistry University of Cambridge Lensfield Road Cambridge CB2 1EW UK; ^2^ UK Dementia Research Institute University of Cambridge Cambridge CB2 0XY UK; ^3^ Department of Molecular Neuroscience Institute of Neurology University College London Queen Square London WC1N 3BG UK

**Keywords:** amyloid fibrils, fluorescence anisotropy, neurodegeneration, Parkinson's disease, protein aggregation

## Abstract

Small aggregates of misfolded proteins play a key role in neurodegenerative disorders. Such species have proved difficult to study due to the lack of suitable methods capable of resolving these heterogeneous aggregates, which are smaller than the optical diffraction limit. We demonstrate here an all‐optical fluorescence microscopy method to characterise the structure of individual protein aggregates based on the fluorescence anisotropy of dyes such as thioflavin‐T, and show that this technology is capable of studying oligomers in human biofluids such as cerebrospinal fluid. We first investigated in vitro the structural changes in individual oligomers formed during the aggregation of recombinant α‐synuclein. By studying the diffraction‐limited aggregates we directly evaluated their structural conversion and correlated this with the potential of aggregates to disrupt lipid bilayers. We finally characterised the structural features of aggregates present in cerebrospinal fluid of Parkinson's disease patients and age‐matched healthy controls.

The formation and spread of small aggregates of proteins such as α‐synuclein, β‐amyloid or tau is reported in a wide range of neurodegenerative diseases.^1^, ^2^ Of these, Parkinson's disease (PD) is characterised by the accumulation of a misfolded and aggregated protein called α‐synuclein within neurons to form Lewy neurites and Lewy bodies.^3^ Genetic and pathological evidence suggests that the protein α‐synuclein is central to neurodegeneration in PD.^4^ Specifically, the transition from an intrinsically disordered α‐synuclein monomer through a series of oligomeric intermediates (with varying structures and size) to a highly structured filament^5^, ^6^ is recognised to drive pathogenesis in α‐synucleinopathies. Furthermore, aggregates of α‐synuclein exhibit cell–cell transfer, leading to seeding and recruitment of more protein molecules to form additional aggregates that can generate new seeds in an exponential way,^7^ leading to the region–region spread of disease. The distinct structure of α‐synuclein aggregates has a role in its pathogenic properties, in particular, the toxicity of the aggregate, the cell type affected, seed competency, and the regional transfer of pathology.^8^, ^9^ This dramatic effect of the structure of the ordered assembly on the pathogenic pathway in the brain underpins the importance of understanding the order/structure of α‐synuclein aggregates.

Previous studies have shown aggregates to be very diverse in terms of their mechanisms of formation, size and structure.^10^, ^11^, ^12^, ^13^ Bulk measurements obtained with conventional, ensemble‐based, biophysical techniques are able to characterise many features of these heterogeneous aggregates,^14^, ^15^ but new quantitative tools are needed to specifically characterise in greater detail the structural features of individual aggregates, particularly in human tissue and biological fluids.

By means of single‐molecule fluorescence resonance energy transfer (smFRET) experiments, a subpopulation of aggregates formed with fluorescently labelled α‐synuclein has previously been shown to undergo a slow structural rearrangement before growing into fibrils.^12^ This conversion can generate more cross‐β structure and the resulting aggregates were reported to be both, more resistant to proteinase‐K and more toxic to cells. Unlabelled fibrils of amyloid‐β or α‐synuclein can be imaged with total internal fluorescence (TIRF) microscopy and structurally specific dyes such as thioflavin T (ThT),^16^, ^17^ opening up the possibilities of studying aggregates in human biofluids.^18^ At a single fibril level, dyes such as ThT or Congo Red have been shown to bind insulin fibrils in an ordered way, and by monitoring the intensity as a function of the polarisation angle, these dye classes can be provide information on the structure of fibrils.^19^

In this work, we have characterised structural features of aggregates formed by an amyloidogenic protein, by implementing a highly sensitive fluorescence anisotropy setup. Fluorophores absorb light with a probability proportional to the square of the dot product of the local optical electric field and the molecular transition dipole moment. Thus when a dye binds in a defined orientation it emits highly polarised anisotropic fluorescence. We therefore built a bespoke anisotropy instrument to study the structure of spatially isolated amyloid aggregates by placing a polariser in the detection path which rotates continuously during image acquisition (Figure 1 a). ThT is a widely‐used benzothiazole dye that increases its fluorescence quantum yield by several orders of magnitude upon binding extended cross‐β structures. Experiments suggest that the dye preferentially binds with its long axis parallel to the long axis of fibrils,^20^, ^21^, ^22^ but depending on the protein under study and the structure of the fibril other binding sites may exist. When single α‐synuclein fibrils are imaged as a function of the angle of the axis of polarisation, their fluorescence modulates sinusoidally between a maximum when the axis is aligned with the fibril and a minimum if the axis is perpendicular to the fibril (Figure 1 b–d). This further confirms that the dominant binding site (or possibly sites) of ThT is aligned with the axis of the α‐synuclein fibrils.

**Figure 1:**
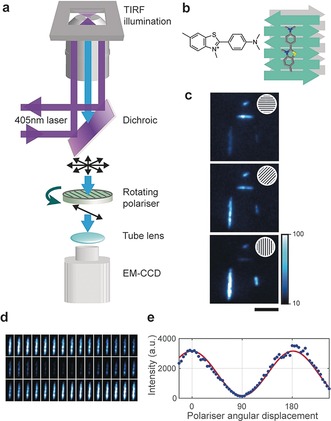
Procedure to monitor anisotropy: a) Fluorescence anisotropy was measured on a total internal fluorescence microscopy (TIRF) arrangement in which a rotating polariser is mounted between the dichroic and the tube lens of the microscope. b) ThT binds specifically to the cross‐β architecture of amyloid fibrils in a well‐defined manner. c) The detected fluorescence of ThT for α‐synuclein fibrils is maximum when the polariser is aligned with the fibril, and minimum when it is orthogonal to the fibril (scale bar=5 μm). d) Montage of the modulating fluorescence of an α‐synuclein fibril as the polariser rotates (each frame corresponds to the intensity averaged during a rotation of approximately 3.6°). e) This intensity can be fitted to a sinusoidal curve to quantify the degree of modulation and hence structural order.

A measure of the degree of extended ordered cross‐β structure in an aggregate is the amplitude of the fluorescence signal of ThT as the polariser rotates. The normalised fluorescence intensity as a function of polariser angular displacement of each individual protein aggregate was fitted to a sinusoid (see Methods section and Figure S1 in the Supporting Information (SI)). The fluorescence was fitted to $y=a\cos\left(bx+c\right)+d$ , where *a* represents the amplitude of the signal, *b* the constant angular frequency (user‐defined rotation velocity of the polariser), *c* the phase and *d* an offset (Figure 1 e). The response to the anisotropy measurement is defined by: modulation=2a/(a+d) . A small modulation value implies disordered β‐sheet content in the aggregate, whereas a larger modulation value implies spatially aligned β‐sheet content.

We used a single‐molecule sensitive TIRF imaging mode to measure the structural arrangement of individual spatially isolated diffraction‐limited aggregates which we have previously characterised using super‐resolution techniques.^23^ We performed an aggregation reaction for recombinant α‐synuclein and focused on the kinetics of the lag phase of the aggregation.^24^ The reaction was done at a concentration of 70 μm under agitation at 200 rpm in 25 mm Tris buffer (pH 7.4) supplemented with 0.1 m NaCl and 0.01 % NaN_3_ at 37 °C. The aggregation reaction was performed in low binding polypropylene tubes to minimise protein adhering to the tubes. More specifically we analysed samples taken from the aggregation reaction at times between 1 h and 4 h. At longer times aggregates larger than the diffraction limit of optical light (i.e. ≈170 nm) start to form. A histogram of modulation values for each time point revealed that oligomers present at 1 h and 2 h have low modulation values (typically lower than 0.5), while at 3 h we found a distinct population of oligomers that respond with high modulation values (Figure 2). These data are indicative of the structural rearrangement from a relatively amorphous oligomer into a “fibril‐like” periodic structure. The modulation measurement does not correlate with the fluorescence intensity of the aggregate (SI, Figure S2 and S3), suggesting that the number of ThT binding sites can remain constant during a structural re‐arrangement. Although there is a variability associated with the stochasticity of the nucleation process during the lag phase of the aggregation, independent experiments show the presence of the two populations of aggregates and the same trend for the evolution of the species (SI, Figure S4).

**Figure 2:**
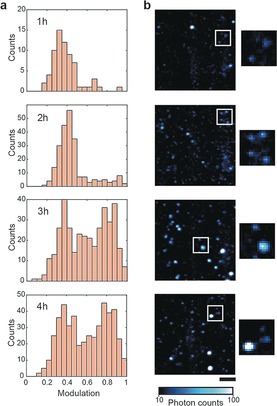
Structural analysis of the species observed during an α‐synuclein aggregation reaction: a) Modulation measurements of detected aggregates during an aggregation reaction at 70 μm show an evolution from relatively disordered aggregates (1 h and 2 h) to a mixture of relatively disordered and “fibril‐like” aggregates (3 h and 4 h). Each histogram pools data from 3 independent aggregations. Representative fluorescence images are shown in (b) (scale bar=5 μm) with magnified sub‐images (2.5x); number of aggregates analysed in each time point: 1 h *n*=121, 2 h *n*=385, 3 h *n*=581, 4 h *n*=719.

Combining all the detected aggregates, the overall distribution of the degree of modulation can be fitted to two Gaussian distributions (Figure 3 a). When compared to the modulation of long fibrils (for example formed after 24 h of aggregation, yielding fibrils that are several μm long), we observe that the fibrils typically have higher modulation values (Figure 3 b). The histogram of oligomers and fibrils can be therefore fitted to three Gaussian distributions (Figure 3 c). The population corresponding to low modulation values have some cross‐β content (as ThT binds to them), and behaves in a similar way to fluorescent beads (SI, Figure S5), meaning that these aggregates are not structurally aligned. The scatter plot of modulation vs. the mean intensity of all measured aggregates suggested once again that disordered aggregates convert to fibril‐like aggregates without an increase in integrated fluorescence intensity (Figure 3 d). We observed that fibrils are characterised by high modulation and intensity values (Figure 3 d). This strong fluorescence anisotropy response of fibrils can be achieved by labelling with other fluorescent dyes such as pentameric formyl thiophene acetic acid (pFTAA) which has also been shown to bind to cross‐β structures^25^ (SI, Figure S6).

**Figure 3:**
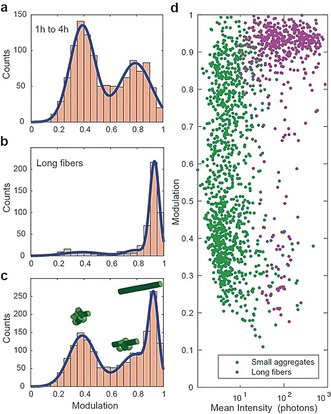
Modulation landscape of small aggregates and fibrils of α‐synuclein: a) Modulation measurements of all species detected in three independent aggregation reactions fitted to two Gaussian functions (1149 species in total). b) Long fibrils (formed after 24 h aggregation, typically several μm in length) respond with high modulation values, which can be fitted adding a third Gaussian (511 species considered). c) The complete landscape of aggregates and fibrils can be fitted with three Gaussian functions. d) Scatter plot of mean intensity of each aggregate (green dots) and fibril (magenta dots) and its corresponding modulation values.

In order to understand further the evolution of aggregates we globally fitted two Gaussian functions to each time point, pooling data obtained in three independent aggregation reactions to describe better the landscape of aggregates (Figure 3 a). The integrated areas of the Gaussians give an estimate of the number of aggregates in each population (Figure 4 a). The results show that non‐modulating aggregates appear before modulating ones, and at a slower rate, suggesting that there is a conversion of non‐modulating to modulating aggregates. In contrast to these well‐defined populations, aggregates formed during an aggregation reaction of α‐synuclein at low monomer concentration (1 μm for 1 month) display a broader distribution of modulation responses, suggesting that a wider variety of species are formed over long periods of time (SI, Figure S7).

**Figure 4:**
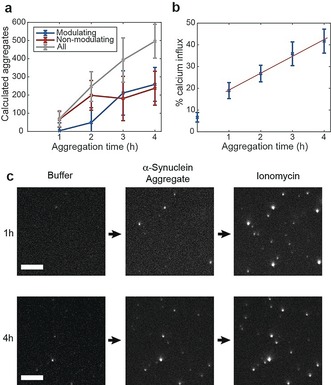
Modulation and toxicity of α‐synuclein during aggregation: a) Number of modulating and non‐modulating α‐synuclein aggregates obtained from the time evolution of the two populations shown in Figure 2 a. (error bars correspond to error in population fits multiplied by the bin size). b) Ca^2+^ influx kinetics obtained with α‐synuclein aggregates (average of the three independent aggregation reactions, error bars are standard deviation between three replicates). c) Example of liposome assay, showing liposomes with disrupted membranes as fluorescent puncta. A higher number of fluorescent liposomes can be seen when incubating with 4 h aggregates as compared with 1 h aggregates (scale bars=5 μm).

To correlate the structural information of the aggregated species with the ability of these aggregates to generate toxic effects in cells we evaluated their capability to disrupt membranes with a technique that uses vesicles filled with a Ca^2+^ sensitive dye.^26^ Upon the interaction of a protein aggregate with the vesicle's membrane, Ca^2+^ ions enter the vesicle from the surrounding solution and hence becomes fluorescent. This change in fluorescence can be detected using TIRF microscopy. We imaged individual liposomes in the presence of Ca^2+^ buffer (blank), followed by the addition of an aliquot of α‐synuclein aggregates at a concentration of 50 nm and subsequent addition of ionomycin. In the presence of only Ca^2+^ buffer, the fluorescence intensity of the vesicles was low and comparable to that of background noise due to minimal Ca^2+^ presence within the vesicle.^26^ After incubation (for 10 minutes) with α‐synuclein samples, we detected an increase in the localised fluorescence intensity of the vesicles showing that Ca^2+^ ions could enter the vesicles as a consequence of the aggregates’ induced membrane permeability. Subsequent addition of ionomycin, an ionophore enabling Ca^2+^ to enter the vesicles, results in the saturation of all vesicles with Ca^2+^ ions, allowing us to quantify the extent of membrane disruption (Figure 4 c).

The permeabilisation assay showed a linear increase of the Ca^2+^ influx after incubation with aggregates (previously aggregated for 1 h to 4 h), suggesting that both disordered and fibril‐like aggregates induce calcium influx in the liposomes (Figure 4 b). Given that the trend of modulating species dominates the later time points while the abundance of non‐modulating aggregates increases at a slower rate (Figure 4 a), our results suggest that modulating species have a higher ability to disrupt membranes.

To demonstrate the broad applicability of our technique, we also applied the method to samples of human cerebrospinal fluid (CSF), comparing aged‐matched healthy controls (HC) to Parkinson's disease (PD) patients. By analysing the modulation of individual species in each group (obtained from 4 HC and 4 PD samples), we found that the large majority of species show non‐modulating behaviour, (Figure 5 a,b) meaning that they are disordered. Only a small fraction (≈1 %) of species showed modulating behaviour in both HC and PD groups (Figure 5 c). Species with higher modulation values (modulation over 0.45) in PD patients have a mean of 0.57 compared to HC with a mean of 0.48 (inset Figure 5 c). The abundance of these modulating species in CSF is very low and therefore prevents us from a robust statistical analysis. Further studies need to be done to characterise these ordered species, as they are candidates to be involved in toxicity and spreading mechanisms. However, this does demonstrate the technique is capable of making structural measurements in human CSF.

**Figure 5:**
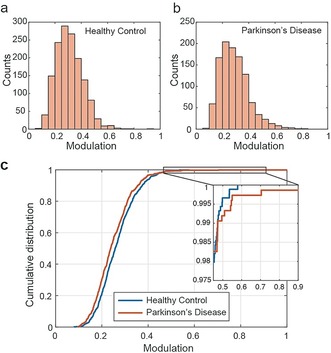
Anisotropy of species observed in human cerebrospinal fluid: Fluorescence anisotropy histogram of species in a) HC CSF and b) PD CSF obtained by pooling aggregates from four different individuals in each case (HC: *n*=1461 species, mean=0.3112, standard deviation=0.1015; PD: *n*=1069 species, mean=0.2929, standard deviation=0.1136). c) Cumulative distribution of species from the same data set showing higher number of modulating species (modulation>0.45) in PD.

In summary, we have demonstrated that by combining sensitive TIRF microscopy with anisotropy measurements, one can directly characterise the structural features of individual oligomers. This method is highly flexible as it does not require protein labelling, but rather a dye that recognises cross‐β motifs. We have shown the conversion from disordered aggregates of α‐synuclein to fibrillar aggregates, in agreement with previously reported smFRET measurements. Furthermore, our experiments suggest that modulating aggregates have a higher capacity to disrupt lipid membranes. Our results provide clear evidence that most ThT active species in CSF are disordered, but do, however, contain cross‐β sheet structure. Our ability to analyse single aggregates individually allowed us to detect an ultra‐low abundance of fibril‐like species in human CSF. This methodology is compatible with other proteins whose aggregation has been associated with human disorders such as amyloid‐β, tau, lysozyme or insulin. Overall this approach provides a new method to characterise the degree of fibrillation in individual protein aggregates, contributing to the set of biophysical methods needed to understand some of the most fundamental mechanisms in neurodegeneration.

## Conflict of interest

The authors declare no conflict of interest.

## Supporting information

As a service to our authors and readers, this journal provides supporting information supplied by the authors. Such materials are peer reviewed and may be re‐organized for online delivery, but are not copy‐edited or typeset. Technical support issues arising from supporting information (other than missing files) should be addressed to the authors.

Supplementary
