# Supplementary material for: Optical Structural Analysis of Individual α‐Synuclein Oligomers
Source: Angew Chem Int Ed Engl. 2018 Mar 23;57(18):4886–90. doi: 10.1002/anie.201710779 (PMC5988047; doi:10.1002/anie.201710779)
Supplement: Supplementary file 1 — Supplementary [file ANIE-57-4886-s001.pdf]

Angewandte Chemie
Eine Zeitschrift der Gesellschaft Deutscher Chemiker

Supporting Information

Optical Structural Analysis of Individual α-Synuclein Oligomers

Juan A. Varela, Margarida Rodrigues, Suman De, Patrick Flagmeier, Sonia Gandhi, Christopher M. Dobson, David Klenerman, and Steven F. Lee**

anie_201710779_sm_miscellaneous_information.pdf

Materials and Methods

Preparation of recombinant α-Synuclein.

Wild-type monomeric α-Synuclein was purified from Escherichia coli as described previously[1]. The protein was concentrated using Amicon Ultra Centricons (Millipore, Billerica, MA), divided into aliquots, before being flash-frozen in liquid nitrogen and stored at −80 °C. Prior to use, the aliquots were ultracentrifuged at 350000g during 1h at 4 °C to minimise the presence of small fibrils, and only the supernatant (containing monomers) was used for aggregation reactions.

Aggregation of α-Synuclein.

For the aggregation reactions, a 70 µM solution of wild-type α-Synuclein in 25 mM Tris buffer (pH 7.4) and 0.1 M NaCl (with 0.01% NaN_3 to prevent bacterial growth during the experiments) was incubated in the dark at 37 °C, with constant agitation at 200 rpm. During incubation, aliquots were taken (at times 1h, 2h, 3h, 4h) and imaged straight away.

Sample preparation for anisotropy measurements.

ThT solutions were prepared as described previously[2]. Stock solutions of ThT (Sigma-Aldrich, product number T3516) were obtained diluting the ThT in ethanol (Sigma-Aldrich, product number 459836) to give a final concentration of ~1 M. This stock was subsequently diluted into PBS (phosphate buffered saline tablets, Sigma-Aldrich, product number P4417) filtered with a 0.02 µm syringe filter (Whatman, product number 6809−2101) to obtain a 50 µM solution of ThT. The final concentration was determined from the absorbance at 412 nm, using an extinction coefficient of 36 000 M^{-1} cm^{-1}. The stock solution was stored in the dark at 4 °C, and was only used for a maximum of 2 weeks after preparation.

Borosilicate glass coverslips (VWR international, 22 × 22 mm, product number 631-0124) were cleaned using an argon plasma cleaner (PDC-002, Harrick Plasma) for at least 1 h to remove any fluorescent residues. Frame-Seal slide chambers (9 × 9 mm, Bio-Rad, Hercules, CA, product number SLF-0201) were affixed to the glass, and 50 µL of poly-L-lysine (70−150 kDa, Sigma-Aldrich, product number P4707-50 ML) was added to the coverslide on the inside of the chamber and incubated for at least 30 minutes and gently washed three times with filtered PBS buffer. Prior to use, each batch of coverslides were tested for fluorescent artefacts (*i.e.* false positives) by analysing a solution of 5 µM ThT only.

Recombinant α-Synuclein samples where diluted into a solution of filtered PBS with 5 µM ThT, to give a final protein concentration of 7 µM. CSF was diluted 10 fold into PBS and ThT was added to obtain a final 5 µM ThT concentration. All samples were stored and diluted in LoBind microcentrifuge (Eppendorf, Hamburg, Germany) to limit surface adsorption.

Imaging.

Imaging was performed using a bespoke total internal reflection fluorescence microscope. For ThT imaging, a 405nm laser (LBX-405-50-CIR-PP, Oxxius) passed through a quarter wave plate (WPQ05M-405, Thorlabs) to obtain a circular polarisation, and then was directed parallel to the optical axis at the edge of a 60× Plan Apo TIRF, NA 1.45 oil-immersion objective lens, (Nikon Corporation); mounted on an Eclipse TE2000-U microscope (Nikon

Corporation) fitted with a Perfect Focus unit. The excitation power was 50 W/cm^2 measured in epifluorescence mode. Fluorescence was collected by the same objective was separated from the returning TIR beam by a dichroic (ZT 405/532 rpc) and passed through an emission filter (FF01-480/40/25).
For pFTAA the excitation was done with a 488nm laser (0488-06-01-0060-100, Cobolt MLD), the dichroic used was Di01-405/488/532/635 (Semrock), and emitted light passed through an emission filter (FF03-525/50-25, Semrock).

The tube lens was removed from the microscope body and placed before the camera, with a separation large enough to fit a polariser (10LP-VIS-B, Newport) mounted on a stepper motor rotation mount (K10CR1/M, Thorlabs) in infinity space between the tube lens and the microscope body. During image acquisition, the polariser was rotated 360 degrees at 2 degrees per second for each field of view. The images were recorded on an EMCCD camera (Evolve 512, Photometrics) operating in frame transfer mode (EM gain of 6.5 e−/ADU and 250 ADU/photon). Each pixel was 241 nm (confirmed by calibration with a Ronchi ruling (600LP per mm). Hardware automation of the microscope was controlled with Micromanager software, and images were recorded at 20 frames per second with an exposure time of 50 ms during a full 360° spin of the polariser.

Image analysis.

Stacks of images corresponding to the full spin of the polariser were denoised in ImageJ (NIH, Bethesda) by sub-averaging 30 frames. As the polariser rotation induces a small circular drift in the images, the stream was aligned using “Align Images” plug-in available in GDSC utility plug-ins from the University of Sussex (http://www.sussex.ac.uk/gdsc/intranet/microscopy/imagej/utility). Once the stack was aligned, an average of the entire stack was generated and used to detect each protein aggregate using the Find Maxima function in ImageJ (with a threshold value of 80). Once the position of each aggregate was recorded, the intensity corresponding to the average of a 3×3 pixels square in each aggregate position was computed. A local background was also calculated for each aggregate, by averaging the intensity along a square of 11 pixels each side, centered in the aggregate (see Supplementary Figure 1). The local background intensity was subtracted to the intensity of each aggregate, and the resulting signal was plotted and fitted with a cosinusoidal curve of the form

$$y = a\cos(bx + c) + d$$

If the quality of the fit was high (checked by visual inspection for every aggregate) the modulation signal was then calculated as follows:

$$modulation = \frac{2a}{a + d}$$

If the phase (*c*) of the aggregate was similar to the phase of the background (within 5°) the aggregate was not considered in the analysis, as we cannot distinguish between a non-modulating aggregate and a modulating one that is oscillating with the same phase as the background.

Membrane permeability assay.

The ability of α-synuclein aggregates to induce membrane disruption was tested with a previously described high-throughput fluorescence intensity based methodology[3]. Vesicles functioning as optochemical probes were tethered to the surface and identical positions of

the glass coverslips were imaged using TIRF microscopy under three different conditions as previously described.

Images were acquired in the presence of only Ca^{2+}-buffer (blank), followed by the addition of an aliquot of α-synuclein aggregates at a concentration of 50 nM and then the addition of ionomycin.

All the TIRF images were acquired with 488nm laser excitation (10 W/cm^2) and averaged over 50 frames with an exposure time of 50 ms each.

References

1. Hoyer, W. *et al.* Dependence of alpha-synuclein aggregate morphology on solution conditions. *J. Mol. Biol.* **322,** 383–93 (2002).
2. Horrocks, M. H. *et al.* Single-Molecule Imaging of Individual Amyloid Protein Aggregates in Human Biofluids. *ACS Chem. Neurosci.* **7,** 399–406 (2016).
3. Flagmeier, P. *et al.* Ultrasensitive Measurement of Ca(2+) Influx into Lipid Vesicles Induced by Protein Aggregates. *Angew. Chem. Int. Ed. Engl.* **56,** 7750–7754 (2017).

Supplementary Figure 1

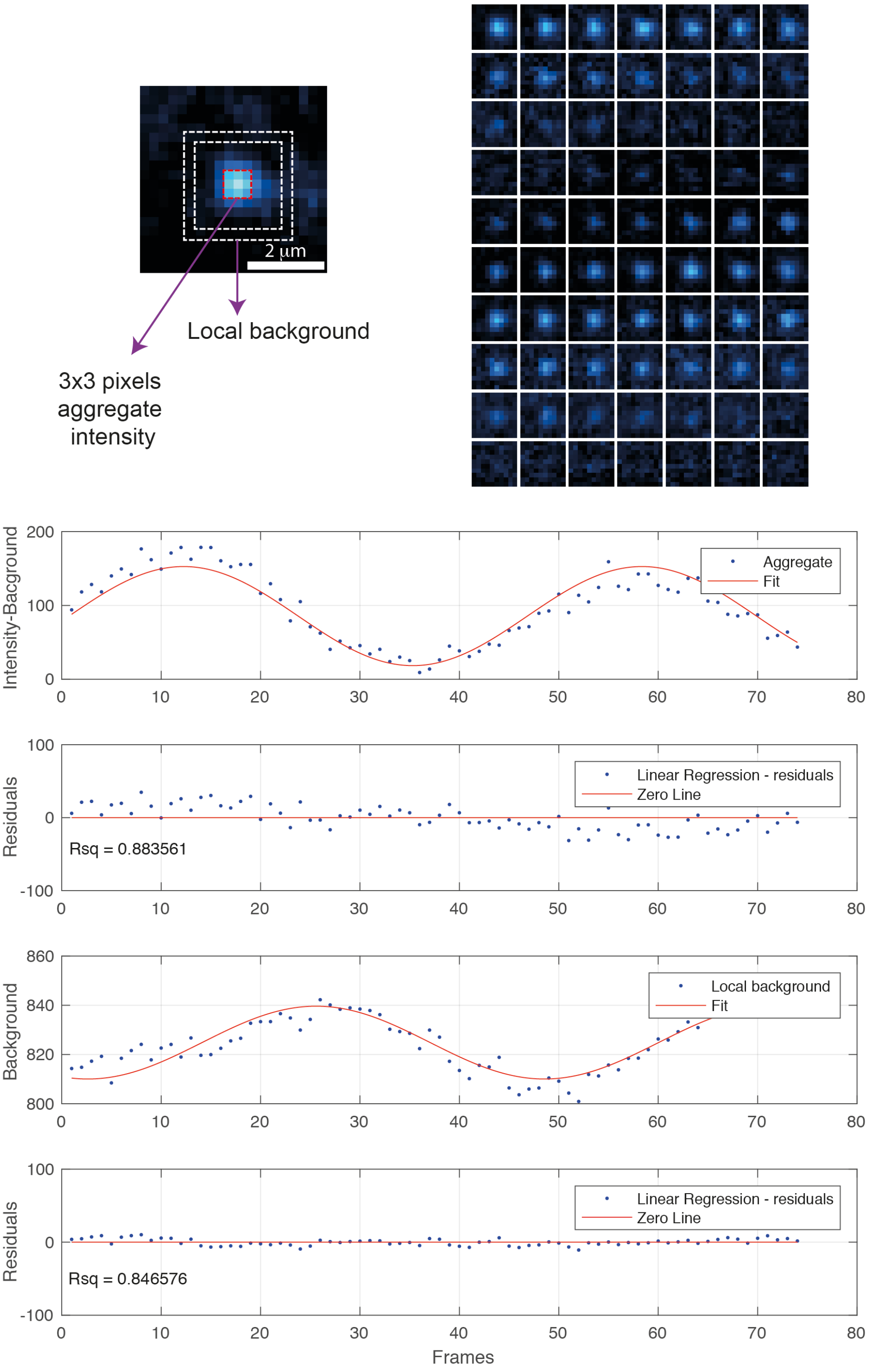

Image of a representative α-synuclein aggregate labelled with ThT, where the selected areas to consider the signal and the background are shown (red and white squares respectively). The variation in intensity as the polariser rotates (illustrated with the montage) is fitted with a sinusoidal function as described in the Methods section, for both the aggregate and the background signal.

Supplementary Figure 2

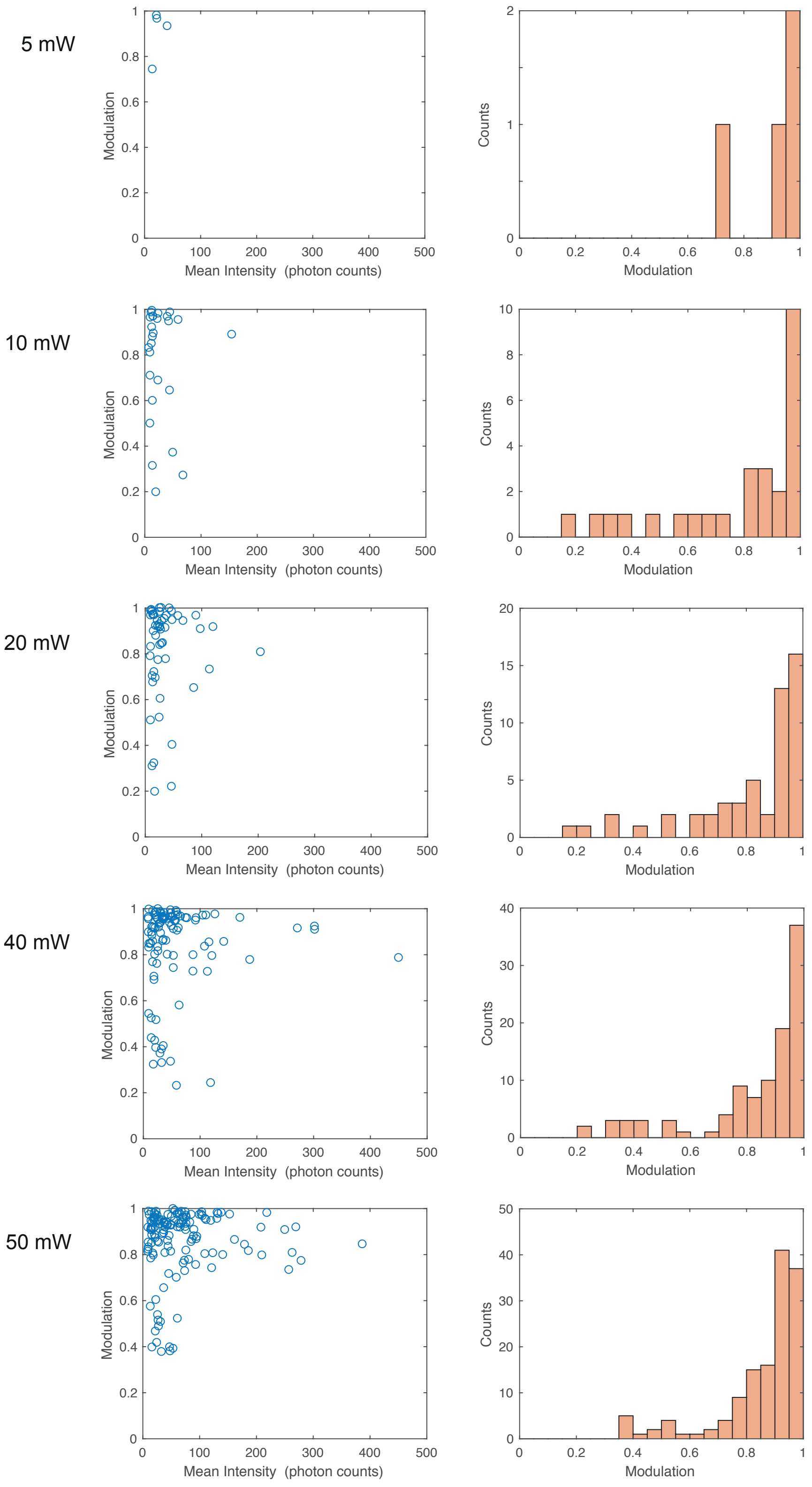

Power dependence of modulation assay over long α-synuclein fibrils. The power of the laser is measured directly at the laser exit.

Supplementary Figure 3

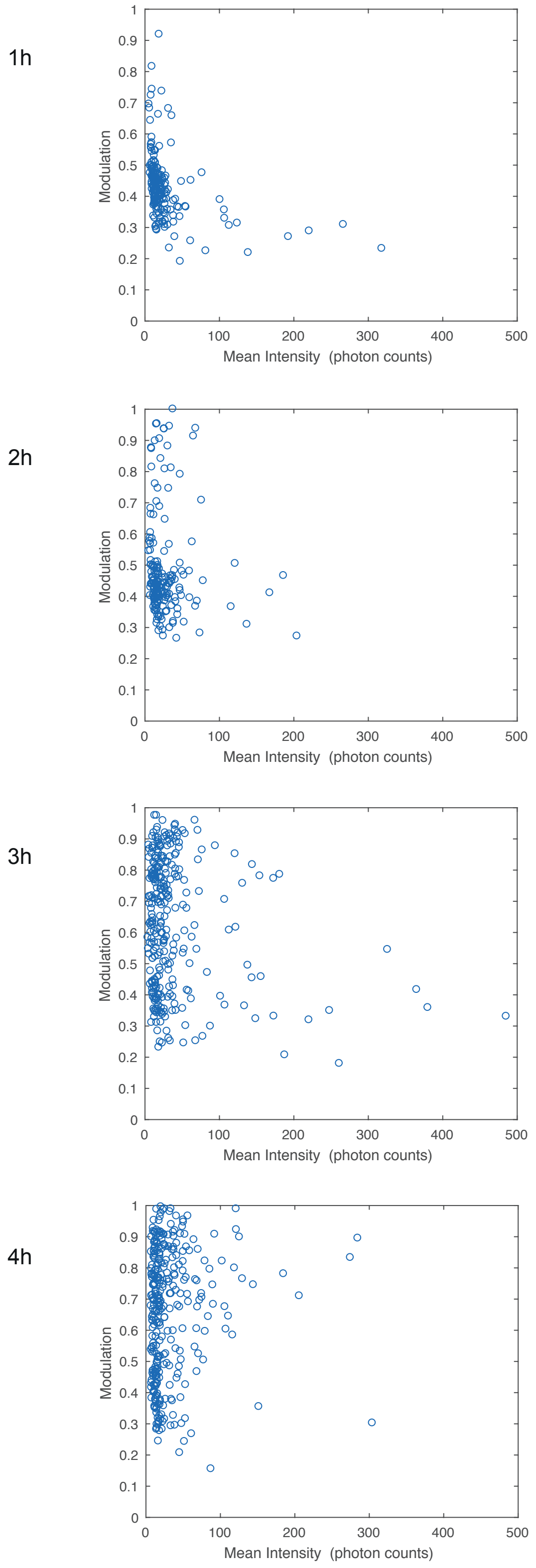

Scatter plots of modulation vs mean intensity of all the aggregates in the histograms of Fig. 2 in the manuscript.

Supplementary Figure 4

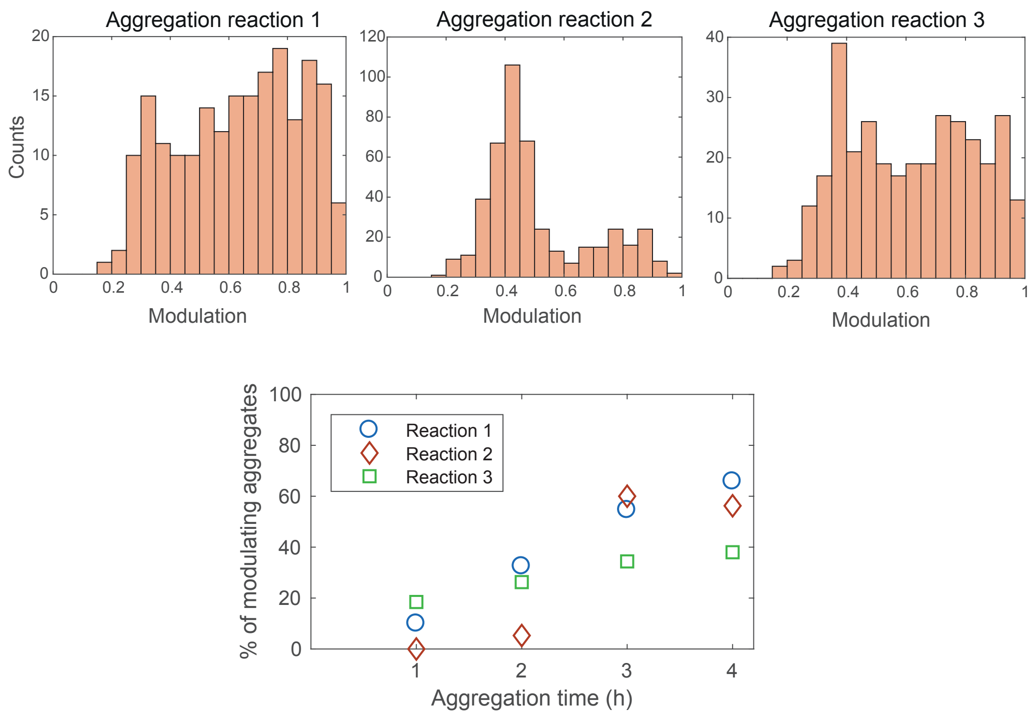

Individual aggregation reactions (pool of 1h to 4h aggregates), all of them showing the present of the modulating and non-modulating populations. Their kinetics of the modulating aggregates (using modulation=0.5 as a threshold for the classification) is shown as a percentage for the three reactions (bottom panel).

Supplementary Figure 5

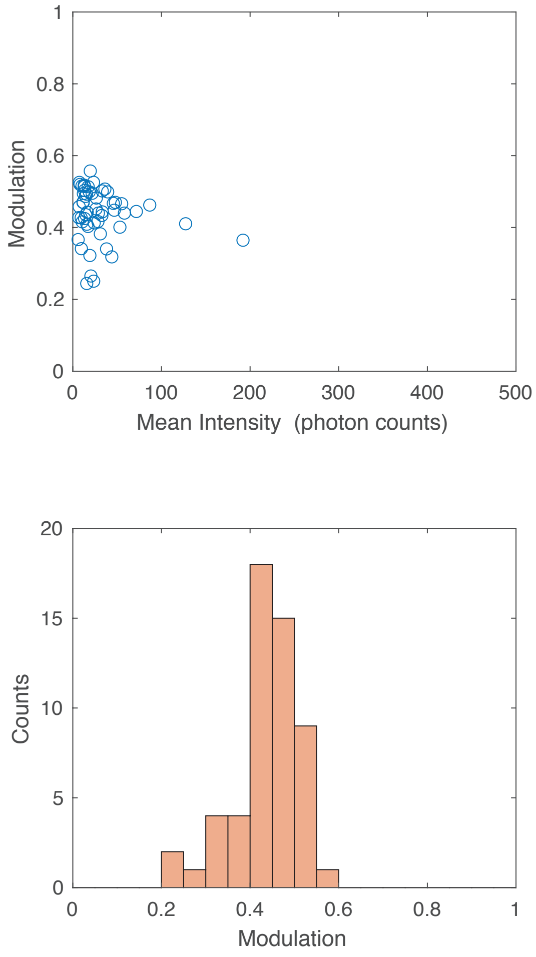

Response of Tetraspeck fluorescent beads (100nm in diameter) to the modulation assay, without ThT in the solution.

Supplementary Figure 6

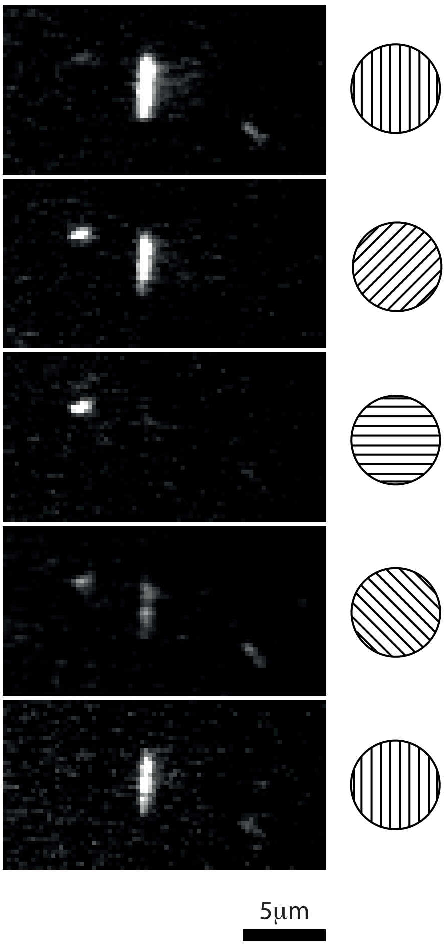

Modulation response of α-synuclein fibrils after 24h aggregation at 70μM using pentameric formyl thiophene acetic acid (pFTAA) at 30nM. The alignment of the polarisation is shown for each frame of the montage.

Supplementary Figure 7

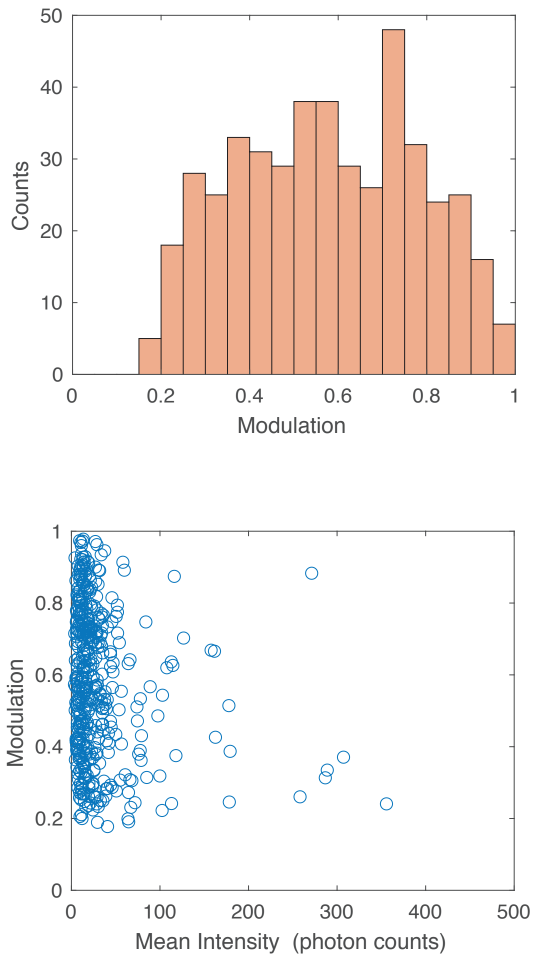

Modulation response of α-synuclein oligomers formed during an aggregation reaction done at a concentration of 1μM for 30 days.
